# Bridging tradition and innovation: electroacupuncture’s impact on premature ovarian insufficiency

**DOI:** 10.3389/fendo.2025.1685306

**Published:** 2026-02-19

**Authors:** Sining He, Lele Ling, Yaran Sheng, Xue Zhao, Long Yuan, Peng Liu, Bingrong Li, Bimeng Zhang

**Affiliations:** 1 Shanghai University of Traditional Chinese Medicine, Shanghai, China; 2 Department of Acupuncture, Shanghai General Hospital, Shanghai Jiao Tong University School of Medicine, Shanghai, China; 3 Department of Obstetrics and Gynecology, Shanghai Sixth People's Hospital Affiliated to Shanghai Jiao Tong University School of Medicine, Shanghai, China; 4 School of Traditional Chinese Medicine, Shanghai University of Traditional Chinese Medicine, Shanghai, China; 5 Department of Geriatrics, Affiliated Longhua Hospital of Shanghai University of Traditional Chinese Medicine, Shanghai, China

**Keywords:** premature ovarian insufficiency, electroacupuncture, traditional Chinese medicine, animal model, mechanism, signaling pathway

## Abstract

Premature ovarian insufficiency (POI) refers to the decline in ovarian function in women before the age of 40, which can lead to premature ovarian failure and ultimately lead to infertility. In recent years, the incidence of POI has continued to rise, posing a serious threat to women’s reproductive health and mental well-being. Although hormone replacement therapy (HRT) is currently the most widely used Western medical treatment method, its long-term use may carry risks such as thrombosis and breast cancer, and it is not yet an ideal treatment option. Electroacupuncture(EA), as an important intervention method in complementary and alternative medicine (CAM), has been shown to exert multisystem regulatory effects, particularly showing promising prospects in the intervention of POI. This review focuses on the mechanism of action of EA in the treatment of POI. First, the advantages and disadvantages of common animal modeling methods were analyzed. The effects of EA have been studied in terms of improving ovarian function, regulating the hypothalamic–pituitary–ovarian (HPO) axis, balancing the neuroendocrine–immune network, alleviating inflammatory responses, regulating local ovarian blood flow, activating mesenchymal stem cell function, and regulating the intestinal microbiota. Research on the mechanism of EA regulation of POI focused on analyzing the phospholipid 3-kinase (PI3K)/protein kinase B (Akt) signaling pathway, Hippo signaling pathway, cell apoptosis, and oxidative stress factors. In addition, this paper summarizes the clinical research progress of different EA treatment regimens in recent years, further verifying their efficacy and safety. Looking ahead, research on EA for POI is expected to achieve breakthroughs with the help of cutting-edge technologies, such as establishing personalized and standardized treatment plans; integrating multidimensional technologies such as genomics, transcriptomics, metabolomics, and tissue clearing; and conducting systematic research on the temporal and spatial dynamic changes in ovarian function. Through the interdisciplinary integration of traditional acupuncture theory and modern life science technology, it is hoped that the underlying mechanisms of EA treatment for POI can be further elucidated, thereby providing a solid theoretical foundation and practical guidance for its clinical application.

## 1 Introduction

Premature ovarian insufficiency (POI) is a common female reproductive endocrine syndrome characterized by ovarian dysfunction before the age of 40, with clinical manifestations including infrequent menstruation, amenorrhea, and abnormal sex hormone levels. Epidemiological data show that among women seeking treatment for infertility, POI and other ovulatory dysfunctions (such as premature ovarian failure and low ovarian response) account for approximately 20% of cases (1). The incidence of POI among women worldwide is approximately 3.5% and continues to rise, becoming a serious public health issue that threatens women’s fertility and physical and mental health (2).

The etiology of POI is complex and involves multiple factors, including genetics, immunity, iatrogenic damage, infection, and living environment (3). Currently, hormone replacement therapy (HRT) is the main clinical treatment. Although it can improve some clinical symptoms, it cannot reverse ovarian damage, and long-term use may increase the risk of adverse reactions such as breast cancer and blood clots (4). In recent years, stem cell therapy has gradually gained attention as an emerging treatment technology. Among them, Mesenchymal stem cells (MSCs) have self-renewal and multidirectional differentiation potential and play a key role in the repair and regeneration of ovarian tissue (5). In addition, vitamin D, as an important factor in regulating the body’s immune system, can effectively reduce malondialdehyde (MDA) levels in ovarian tissue, playing an important role in anti-inflammation and immune regulation (6). However, the aforementioned methods are limited in their widespread application due to high costs, technical barriers, and unclear mechanisms of action. Therefore, the development of safe, effective, and mechanism-clear alternative or adjunctive therapeutic approaches has become a key focus of current research.

Electroacupuncture (EA), as an intervention method that combines traditional Chinese acupuncture with modern electrical stimulation technology, shows great potential for application in many disease areas. A multicenter randomized controlled study involving 504 female subjects showed that EA can promote the reinnervation of pelvic floor muscles and increase muscle strength, thereby effectively relieving the symptoms of stress urinary incontinence (7). In addition, EA can also weaken insulin resistance caused by autophagy defects in rats with polycystic ovary syndrome (PCOS) by inhibiting the mTOR/4E-BP1 signaling pathway (8). In POI intervention studies, a large body of experimental evidence has shown that EA can exert ovarian protective effects through multiple mechanisms, including regulating signaling pathways, improving the ovarian microenvironment, regulating sex hormone levels, and protecting granulosa cell function. For example, EA can regulate the secretion levels of key hormones such as follicle-stimulating hormone (FSH), estrogen (E2), and anti-Müllerian hormone (AMH) and intervene in the apoptosis of granulosa cells and the autophagy process of mitochondria, thereby achieving the restoration of ovarian function (9). Clinical studies have also shown that EA treatment for POI has an overall efficacy rate of up to 95%, which is significantly better than HRT, with a lower incidence of adverse reactions, good safety, and good patient compliance (10).

In recent years, studies have suggested that gut microbiota diversity is involved in estrogen metabolism regulation and that there is a bidirectional regulatory relationship between gut microbiota and steroid hormones, known as the “gut–ovary axis”. Specifically, gut microbiota can influence ovarian function, and conversely, steroid hormones can also feedback-regulate the composition of gut microbiota (11, 12). Previous studies have confirmed that EA can improve ovarian function by increasing the abundance and diversity of beneficial bacteria in the gut, thereby further validating the role of the “gut–ovary axis” in the pathogenesis of POI (13). At the same time, although AMH is currently one of the main biomarkers for assessing ovarian reserve function, its predictive accuracy improves with age, so its predictive value for young women remains limited (14). To this end, researchers are constantly exploring new biomarkers. For example, oxidative stress-induced mitochondrial DNA (mtDNA) mutations can accumulate in the ovaries, accelerating tissue aging, whereas microRNA (miRNA) expression patterns differ among women of different ages, providing a new molecular perspective for elucidating ovarian function decline (15). Previous studies have found that EA applied to the “Zhongliao (BL33)” and “Tianshu (ST25)” acupoints can downregulate the expression of insulin-like growth factor 1 receptor (IGF-1R) mRNA in ovarian tissue, thereby improving ovarian function and providing an experimental basis for the widespread application of new biomarkers (16).

In summary, the mechanism of action and clinical value of EA in POI treatment are receiving increasing attention, and related research is becoming increasingly systematic and in-depth. This paper aims to comprehensively review the recent progress in basic and clinical research on EA treatment for POI, with a focus on summarizing research findings in areas such as mechanism of action, animal model selection, and optimization of clinical protocols. It further explores potential future directions for breakthroughs, with the goal of providing theoretical foundations and practical guidance for EA intervention in POI.

## 2 Western medical understanding of POI

### 2.1 Definitions

**Figure 1:**
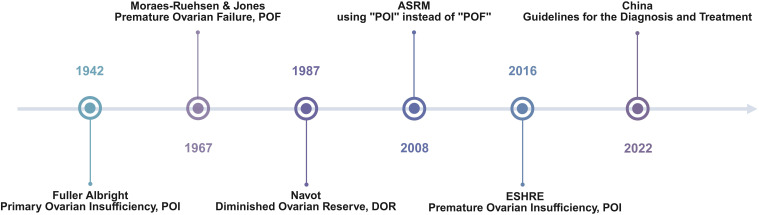
Evolution of terminology related to premature ovarian insufficiency.

POI refers to the decline in ovarian function in women before the age of 40, characterized by infrequent menstruation or amenorrhea, accompanied by elevated FSH levels and decreased E2 and AMH levels, constituting a clinical syndrome (17). It is worth noting that it took 70 years for the name POI to be finally established. Since Albright first proposed “primary ovarian insufficiency” in 1942, the name of the disease has undergone several changes. In 1967, Moraes-Ruehsen and Jones proposed the term "premature ovarian failure (POF)", whereas Navot introduced the term “diminished ovarian reserve (DOR)". It was not until 2016 that the European Society of Human Reproduction and Embryology (ESHRE) formally established “premature ovarian insufficiency (POI)” as the standard term in its guidelines, emphasizing that the process involves gradual functional decline rather than complete failure. In China, the Guidelines for the Integrated Traditional and Western Medicine Diagnosis and Treatment of Premature Ovarian Insufficiency were issued in 2022 by the Obstetrics and Gynecology Professional Committee of the Chinese Society of Integrated Traditional and Western Medicine, providing the most authoritative national standard for clinical diagnosis and treatment (**Figure 1**).

#### 2.1.1 Premature ovarian insufficiency and premature ovarian failure

Premature ovarian failure (POF) typically refers to the complete loss of ovarian function in women before the age of 40, manifested by amenorrhea, significantly reduced estrogen levels, and significantly elevated FSH levels (>40 IU/L). POF emphasizes the “terminal state” of the ovaries, and patients often find it difficult to naturally restore ovarian function. POI, on the other hand, emphasizes the reversibility of the process of ovarian function decline, and some patients may still experience intermittent ovulation or natural pregnancy. To accurately reflect the progressive nature of this condition, ESHRE renamed “POF” to “POI” in its 2016 guideline, “ESHRE Guideline: Management of Women with Premature Ovarian Insufficiency”, and lowered the diagnostic threshold for FSH from 40 to 25 IU/L (18). This change not only facilitates early identification of ovarian dysfunction but also aligns with the traditional Chinese medicine concept of “Preventing a disease before it arises”.

#### 2.1.2 Premature ovarian insufficiency and decreased ovarian reserve function

Diminished ovarian reserve (DOR) refers to a decrease in the number and quality of follicles in the ovaries, leading to reduced fertility potential, but not yet progressing to the POI stage. The diagnostic criteria for DOR mainly include AMH level <1.1 ng/mL, FSH >10 IU/L, and antral follicle count (AFC) <5–7 (19). DOR refers to early decline in ovarian reserve function. Menstrual cycles may be normal, but in older women (≥35 years old), if pregnancy has not been achieved after more than 6 months of continuous attempts, an ovarian reserve assessment should be conducted. DOR is an early stage of POI development, and if left untreated, it may gradually progress to POI. Therefore, clinicians should pay close attention to DOR and identify and treat it early.

### 2.2 Etiology

**Figure 2:**
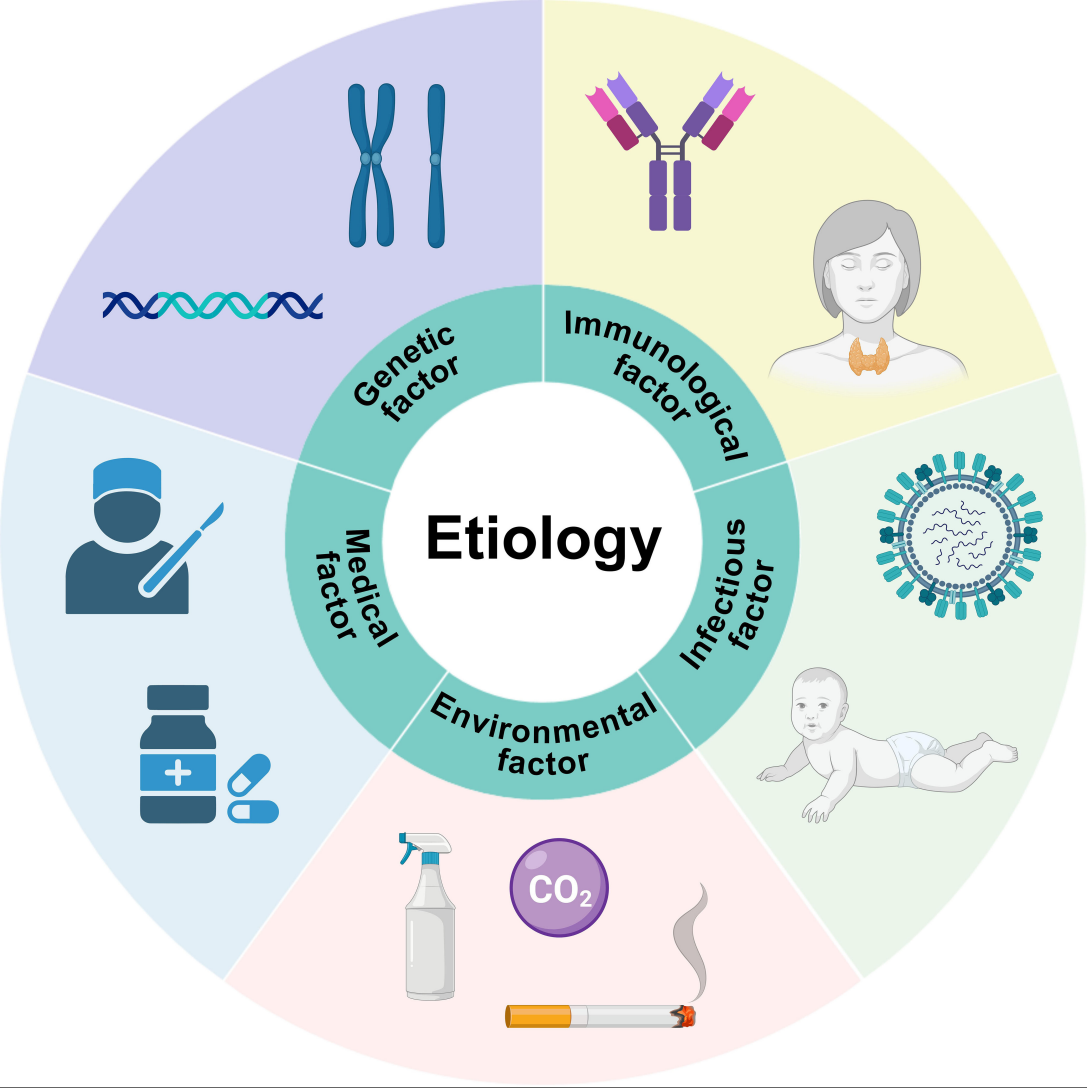
Common causes of POI.

The etiology of POI is complex and multifactorial, involving genetic, autoimmune, iatrogenic, infectious, and environmental factors (**Figure 2**). These diverse mechanisms may converge on common pathological pathways such as oxidative stress, immune dysregulation, and epigenetic alteration, ultimately leading to accelerated follicular depletion and ovarian atrophy.

#### 2.2.1 Genetic factors

Genetic factors are one of the important pathogenic mechanisms of POI, including chromosomal abnormalities and gene mutations (20). A Dutch research report shows that 12% to 50% of POI patients have high genetic heterogeneity, which may be due to genes and their interaction with the environment (21). In POI patients, X chromosome abnormalities are more common than autosomal abnormalities, including structural variations such as X chromosome deletions, duplications, and translocations (22). Turner syndrome (TS) is one of the most common X chromosome abnormalities, with a typical karyotype of 45, X. Patients often experience primary amenorrhea before puberty and early follicular depletion, resulting in impaired secondary sexual characteristic development and infertility (23). In addition, among carriers of fragile X syndrome caused by FMR1 gene mutations, the incidence of POI is as high as 24% (24). In recent years, genetic analysis of POI patients using whole-exome sequencing (WES) has revealed that mutations in genes such as NR5A1 and MCM9 are relatively common in patients, involving meiosis and DNA repair mechanisms (25). These findings expand the molecular spectrum of POI and provide new avenues for targeted therapeutic strategies.

#### 2.2.2 Autoimmune factors

Approximately 20% to 30% of POI patients also have autoimmune diseases, suggesting that immune mechanisms play an important role in the development of POI (26, 27). In some patients, the body’s immune system mistakenly identifies ovarian tissue as a foreign antigen, producing anti-ovarian antibodies that cause ovarian inflammation and damage, reducing the number of follicles and causing ovarian stromal fibrosis, leading to ovarian dysfunction (28). Common comorbidities include autoimmune thyroid disease (AITD), systemic lupus erythematosus (SLE), Addison’s disease, and rheumatoid arthritis (29). Shared genetic susceptibilities and cytokine dysregulation may underlie these overlapping autoimmune mechanisms.

#### 2.2.3 Iatrogenic factors

Iatrogenic factors are also an important cause of POI, especially in women who have undergone reproductive system surgery, radiotherapy, or chemotherapy. It is reported that patients who underwent oophorectomy developed POI symptoms on average within approximately 6 years (30). In addition, radiotherapy can cause radiation damage at different stages of follicle development, especially in resting primordial follicles, which are most sensitive to radiation and highly susceptible to apoptosis (31). For women under 40, a dose of 20 Gy may cause ovarian failure; for older women, a treatment dose of only 6 Gy can cause permanent infertility (32). Among chemotherapy drugs, alkylating agents are the most toxic to the ovaries, significantly reducing the number of follicles and particularly affecting the reproductive potential of children and adolescent patients (33).

#### 2.2.4 Infectious factors

Infectious factors play an important role in the pathogenesis of POI. In addition to the well-known mumps, tuberculosis, and chickenpox, other viral infections such as cytomegalovirus (CMV), rubella virus, and HIV have also been shown to be associated with ovarian dysfunction (34). Oophoritis caused by mumps virus infection is considered one of the potential causes of idiopathic POI. The mechanism may involve direct viral invasion or the induction of an autoimmune response, ultimately leading to follicular atresia and functional loss (32). Clinical studies have found that approximately 3%-7% of POI patients have detectable mumps virus antibodies in their serum, and there is a direct correlation between the presence of these antibodies and a decline in ovarian reserve (35). It is worth noting that recent studies have found that the novel coronavirus (SARS-CoV-2) can bind to the angiotensin-converting enzyme 2 (ACE2) receptor, leading to viral invasion of reproductive cells and resulting in reduced fertility in women (36).

#### 2.2.5 Environmental factors

Endocrine-disrupting chemicals (EDCs), which are prevalent in the modern environment, are closely related to the onset of POI. Phthalates can inhibit follicle growth by reducing the levels of aromatase mRNA and protein in granulosa cells, whereas high doses of bisphenol A (BPA) can reduce serum E2 levels and inhibit oocyte maturation (37). PM2.5, NO2, and other pollutants present in the air can enter the body through the respiratory tract or food, interfering with the endocrine system. There is a clear negative correlation between air pollution levels and serum AMH levels (38). Tobacco exposure is also an independent risk factor. Nicotine metabolites can reduce ovarian antioxidant capacity and accelerate follicle apoptosis (39). In addition, heavy metal pollution should also be taken seriously. One study found that rats exposed to environments containing 5–50 μM of cadmium experienced low DNA methylation in the SCF/c-kit promoter region, which in turn inhibited follicle development (40).

### 2.3 Clinical manifestations and diagnosis

The typical clinical manifestations of POI are menstrual disorders, including infrequent menstruation, prolonged cycles, reduced menstrual flow, and even amenorrhea. Decreased estrogen levels can trigger perimenopausal symptoms such as hot flashes, night sweats, mood swings, insomnia, and vaginal dryness. Some patients may experience underdeveloped secondary sexual characteristics or decreased libido. In addition, POI is also associated with osteoporosis, cardiovascular disease, lipid metabolism abnormalities, and cognitive decline, which severely affect patients’ quality of life and long-term health (41).

The diagnostic criteria for POI are based on the latest expert consensus from 2023 (17); the main criteria include the following: women who experience menopause or infrequent menstruation for at least 4 months before the age of 40, with serum FSH levels greater than 25 IU/L, measured at least twice with an interval of 4 weeks between measurements. Clinical evaluation should include medical history collection, physical examination, hormone level measurement, and auxiliary examinations. It is necessary to focus on identifying relevant causes, such as chromosomal abnormalities, autoimmune diseases, and pituitary dysfunction. Imaging (such as ultrasound) can be used to assess ovarian volume and antral follicle count (AFC). If necessary, anti-ovarian antibody testing, thyroid function screening, and genetic testing can also be performed.

### 2.4 Treatment methods

Currently, western medicine offers a variety of therapeutic approaches targeting hormone restoration, follicular regeneration, and fertility preservation. HRT remains the cornerstone of treatment. However, it does not restore ovarian reserve. Emerging regenerative strategies—such as stem cell transplantation, exosome therapy, *in vitro* activation (IVA), platelet-rich plasma (PRP) injection, and mitochondrial transfer—have shown promise in preclinical or early clinical studies. Complementary and alternative medicine (CAM) are also being investigated for symptom management and quality-of-life improvement. Fertility preservation techniques, including oocyte and embryo cryopreservation, offer reproductive options for women at risk of POI. The specific applications and limitations of these treatments are summarized in **Table 1**.

**Table 1:** Western medical treatment methods and limitations.

Treatment methods	Specific applications	Main limitations	References
Hormone replacement therapy (HRT)	Exogenous estrogen/progesterone supplementation to relieve symptoms and delay menopause-related complications.	Ovulation cannot be restored; long-term use may increase the risk of breast cancer, blood clots, etc.	(129–131)
Stem cell therapy	Transplantation of autologous or allogeneic MSCs to promote ovarian tissue repair.	High costs, unclear differentiation mechanisms, ethical controversies, and technical obstacles.	(132–134)
Exosome therapy	Application of MSC-derived exosomes to regulate inflammatory, antioxidant, and antiapoptotic pathways.	Low delivery efficiency, safety, and long-term efficacy have not been adequately verified.	(135–137)
*In vitro* activation therapy (IVA)	Activate signaling pathways in ovarian tissue to awaken dormant follicles.	Signal transduction pathways have complex mechanisms of action and imprecise regulation, which may lead to abnormal follicle development.	(138–140)
Platelet-rich plasma therapy (PRP)	Injecting autologous platelet-rich plasma improves local blood flow, increases growth factor release, and promotes follicle development.	The effectiveness varies greatly among individuals, and some studies show that the improvement in AMH is not significant.	(141–143)
Mitochondrial activation and transfer therapy	Use drugs to restore mitochondrial function and use transfer technology to replace defective mitochondria.	The research is in the experimental stage and lacks clinical trials to verify its safety and efficacy.	(144)
Complementary and alternative medicine (CAM)	Including cognitive behavioral therapy, calcium/vitamin D/vitamin E supplementation, dietary intervention, and exercise management.	There is a lack of high-quality RCTs, and the specific mechanism is unclear; it relies heavily on patients’ self-management abilities.	(145)
Assisted reproductive technology	Egg donation, *in vitro* fertilization, ovarian tissue freezing and transplantation, etc.	High cost, some approaches involve ethical/legal issues; success rates are significantly affected by the condition of the ovaries.	(146–148)

## 3 Traditional Chinese medicine’s understanding of POI

**Figure 3:**
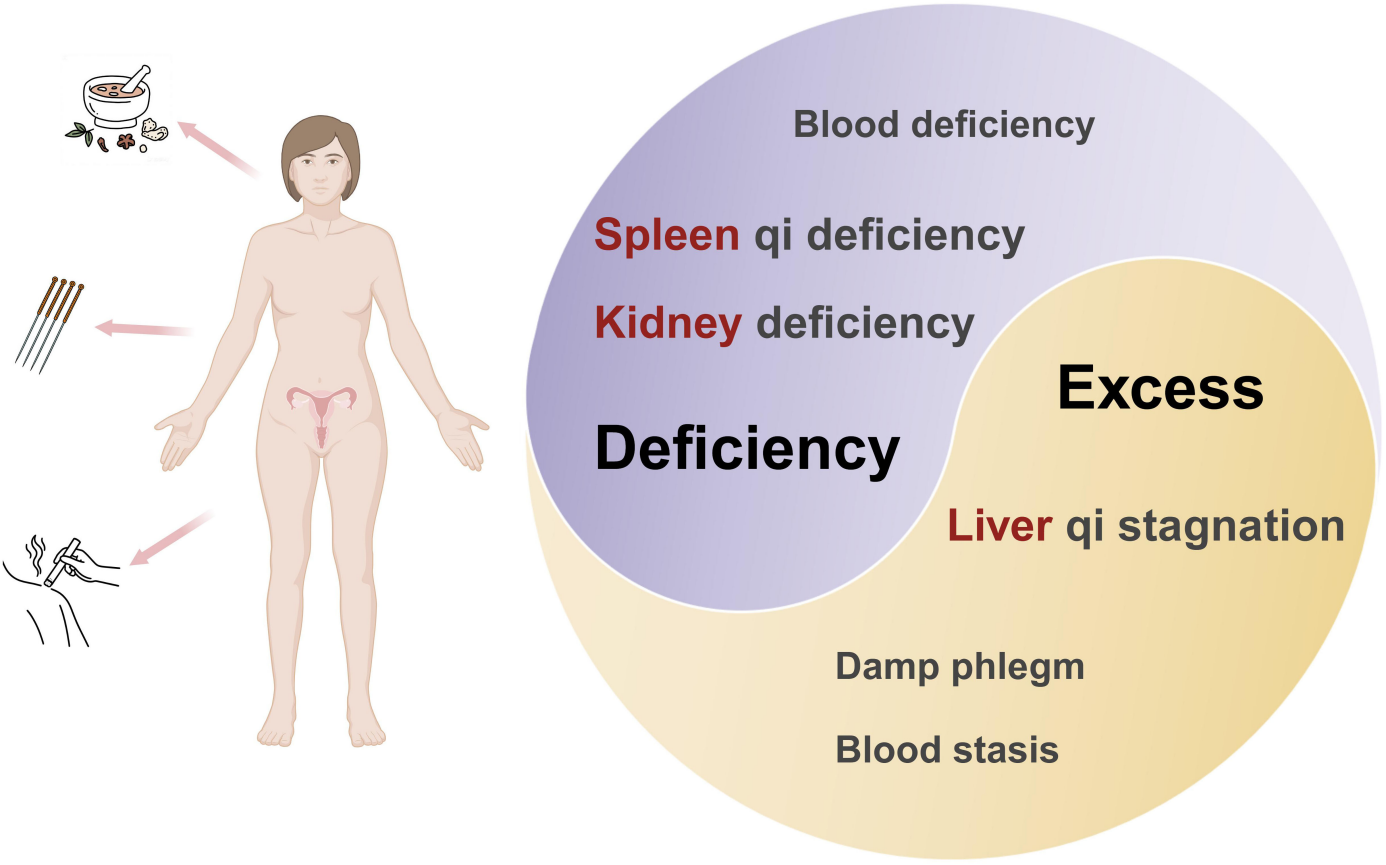
Traditional Chinese medicine’s understanding of POI.

POI does not have a clear corresponding disease name in traditional Chinese medical texts, and its symptoms can be classified under categories such as “amenorrhoea,” “infertility,” and “menstrual disorders”. Suwen • Shang Gu Tian Zhen Lun records: “At the age of 49, the Ren meridian becomes deficient, the Tai Chong meridian declines, the Tian Gui dries up.” It refers to the cessation of menstruation in women at the age of 49 due to the depletion of the menstrual blood. If a woman experiences premature menopause before reaching old age, it is considered pathological premature aging and meets the modern definition of POI. Traditional Chinese medicine believes that the occurrence of POI is closely related to “congenital deficiency, acquired depletion, and emotional injury,” involves dysfunction of multiple organs such as the kidneys, liver, and spleen, and often presents with symptoms characteristic of “combined deficiency and excess” (42). Modern research has gradually integrated with traditional Chinese medicine theory, providing multidimensional support for a deeper understanding of the etiology and pathogenesis of POI (**Figure 3**).

### 3.1 Etiology and pathogenesis

The kidney is the foundation of innate constitution and govern reproduction. Congenital deficiency is one of the important causes of POI. Women are defined by the number “seven”. Suwen: Shang Gu Tian Zhen Lun points out that the kidneys play a leading role in the production and cessation of menstruation and that the abundance of kidney essence is directly related to female reproductive function. When kidney qi is strong, the menstrual cycle begins; when kidney qi is weak, the menstrual cycle ceases. Menstrual blood is a reproductive substance generated from kidney essence. Insufficient congenital foundation leads to insufficient menstrual blood production, which affects female reproductive function. The Chong Meridian is the sea of blood, regulating the qi and blood of the 12 meridians. The Ren Meridian is the sea of yin meridians, regulating the qi and blood of the yin meridians. When the qi and blood of the Chong and Ren meridians are abundant, the qi and blood in uterus is full, the ovaries function properly, and menstruation occurs regularly. Subsequent generations developed the “Kidney-Tian Gui-Chong-Ren-Uterus Axis” theory, emphasizing that the abundance of kidney essence is the foundation of female reproductive function. Any disruption in any link of the axis can lead to the depletion of Tian Gui before the age of 49 (43).

Acquired depletion is also a significant cause of POI. Long-term irregular eating habits or picky eating can damage the spleen and stomach, leading to insufficient production of qi and blood, which in turn fails to nourish the ovaries. The spleen is the foundation of postnatal nourishment and the source of qi and blood production, which in turn nourishes the innate essence. Tian Gui originates from the essence and qi of the kidneys and requires nourishment from the essence of postnatal food and drink to function normally. Therefore, when the spleen and stomach are healthy, Tian Gui is abundant, the uterus is nourished, and menstruation is regular (44).

Emotional injury is particularly common among modern women. Related studies have shown that the prevalence of anxiety and depression symptoms among POI patients is significantly higher than that of the general population (45). Long-term estrogen deficiency can lead to endocrine disorders, causing symptoms such as hot flashes, sweating, irritability, insomnia, and fatigue. These symptoms contribute to the subjective physical sensations experienced by patients with POI. Prolonged physical discomfort and psychological stress can severely impair patients’ quality of life, further exacerbating endocrine disorders within the body and triggering a series of negative emotions, thereby creating a vicious cycle (46). The liver is responsible for regulating the flow of qi. When liver qi flows smoothly, blood circulation is unimpeded. However, when emotions are not properly expressed, liver qi becomes stagnant, leading to imbalances in the Chong and Ren meridians, menstrual irregularities, abnormal accumulation or overflow of blood in the uterus, and menstrual blood failing to flow as scheduled. Ling Shu states, “Grief, sorrow, and worry disturb the Heart”. Emotional distress can affect the heart and spleen and, over time, lead to deficiency of both qi and blood, causing the uterus to become malnourished.

In addition, pathological products such as blood stasis and phlegm dampness obstructing the cell membranes are also causes of disease (47). Stagnation of blood and qi in the collaterals, impaired circulation of qi and blood, and disharmony of the Chong and Ren meridians lead to inadequate nourishment of the ovaries, resulting in premature aging. Stagnant blood and phlegm fluid are pathological products of the body that obstruct the circulation of qi and blood, causing stagnation in the collaterals. When qi, blood, and body fluids cannot be distributed properly, the essential nutrients required to nourish the uterus gradually diminish, and over time, disease arises (48).

In summary, POI is mostly characterized by deficiency and excess. The deficiency is mainly kidney deficiency, accompanied by spleen deficiency and blood deficiency, whereas the excess is commonly liver depression, phlegm dampness, and blood stasis. Its pathogenesis is characterized by multiple causes and mechanisms coexisting and interacting with each other.

### 3.2 Treatment methods

#### 3.2.1 Traditional Chinese medicine treatment

Traditional Chinese medicine treatment for POI iscentered on syndrome differentiation and treatment. Common syndromes include kidney qi deficiency, kidney yin deficiency, spleen–kidney deficiency, and liver qi stagnation with blood stasis. Based on the syndrome type, deficiency syndromes are treated with tonification, such as tonifying the kidneys to replenish essence, tonifying the spleen to strengthen qi, or nourishing blood to replenish yin, to nourish the source of menstrual blood. Excess syndromes are treated with drainage, such as regulating the liver and harmonizing qi, or promoting blood circulation and resolving stasis, to facilitate the smooth flow of qi and blood in the collaterals. For example, Si Wu Tang can regulate qi and blood, improve ovarian granulosa cell apoptosis, and exert antiapoptotic and antioxidant effects (49). Danggui Shaoyao San exhibits protective effects on the ovaries by positively regulating protein phosphorylation and oxidative stress responses and inhibiting apoptosis pathways (50). Kuntai Capsules contain components such as paeoniflorin and malic acid, which can regulate molecular targets such as FOXO3 and SIRT5 to improve ovarian function (51).

The concept of treating conditions in stages is another distinctive feature of traditional Chinese gynecology. The continuous transformation between yin and yang forms the menstrual cycle of women. The menstrual period, post-menstrual period, intermenstrual period, and premenstrual period correspond to the sequential transformations of “heavy yang transforming into yin,” “yin growing while yang diminishes,” “heavy yin transforming into yang,” and “yang growing while yin diminishes”. Stage-based treatment follows the changes in the abundance and deficiency of qi and blood during the menstrual cycle, selecting appropriate methods such as nourishing blood and regulating menstruation, tonifying the kidneys and enhancing essence, and soothing the liver and alleviating depression for treatment. For example, Professor Zhao Runze and others used Zuo Gui Wan and You Gui Wan alternately according to the menstrual cycle (52). Professor Wang Yanan emphasized that “tonifying the kidneys and regulating the menstrual cycle” fully leverages the advantages of integrated traditional Chinese and Western medicine treatment, addressing both the symptoms and root causes (53).

#### 3.2.2 Acupuncture treatment

Acupuncture therapy, as an important component of traditional Chinese medicine, is widely used in the treatment of POI. The selection of acupoints is mainly based on replenishing vital energy, regulating the Chong and Ren meridians, and promoting blood circulation (54). Common primary acupoints include Guanyuan (CV4), Shenshu (BL23), Sanyinjiao (SP6), Zusanli (ST36), and uterus (EX-CA1), supplemented by acupoints that promote liver qi regulation or spleen qi tonification. Professor Yang Zhuoxin proposed the “Regulating the Ren and Du Meridians” acupuncture technique, which harmonizes the Ren and Du meridians to achieve yin–yang balance and orderly Chong and Ren meridian function, thereby promoting the recovery of ovarian function (55). Other studies have shown that transcutaneous electrical nerve stimulation (TENS) therapy can improve pathological changes in ovarian tissue, reduce interstitial fibrosis, increase the number of well-developed corpus luteum and intact primordial follicles, and raise serum AMH levels, thereby delaying the progression of ovarian damage (56).

#### 3.2.3 Integrative medicine treatment

The combination of traditional Chinese medicine and Western medicine for the treatment of POI is increasingly becoming the mainstream clinical treatment method. Research shows that the combination of traditional Chinese medicine and HRT can regulate serum hormone levels, synergistically improve ovarian function, reduce the incidence of side effects from Western medicine, significantly shorten the dosage and duration of hormone therapy, and improve patient compliance (57). The use of bone marrow mesenchymal stem cells (BMSCs) to treat POI is also a hot topic of research today. Acupuncture combined with MSCs has also been shown to enhance stem cell homing ability and effectively reduce excessive activation of mitochondrial autophagy, preventing mitochondrial damage and exerting a stronger ovarian repair effect (58).

#### 3.2.4 Others

Traditional Chinese medicine also offers a variety of treatment methods, such as herbal paste formulas, acupoint implantation, and herbal enemas. Herbal paste formulas are convenient to carry and excel in nourishing the body; acupoint implantation is another highlight in acupuncture therapy, involving the insertion of absorbable protein threads into acupoints, combining the effects of acupuncture, sustained stimulation, and immune regulation. These methods have demonstrated good results in improving patients’ symptoms and immune indicators (59, 60). Traditional Chinese medicine also emphasizes lifestyle adjustments, including emotional balance and moderate exercise, to strengthen the body and prevent disease.

## 4 Mechanism study of electroacupuncture treatment for POI

### 4.1 Animal model research

Animal experiments form the foundation of basic research, making the selection of animal modeling methods particularly important. The table below summarizes the commonly used animal modeling methods for POI in experimental research, along with their specific applications and advantages and disadvantages. These methods primarily include chemical induction, gene editing, immune induction, radiation induction, environmental toxin induction, dietary induction, stress induction, surgical induction, and natural aging models (**Table 2**).

**Table 2:** Advantages and disadvantages of laboratory molding methods.

Molding method	Specific applications	Principle	Advantages	Disadvantages	References
Chemical induction method	Injection of drugs such as cyclophosphamide, busulfan, cisplatin, and Tripterygium glycosides	Induce DNA damage or early activation and apoptosis in follicles through chemical drugs.	Easy to operate, low cost; the animal model established is more stable.	Drug toxicity may affect other organs; model stability is poor, and there are large individual differences.	(149)
Gene editing method	CRISPR/Cas9 technology	Knock out or mutate genes related to ovarian function, such as FOXL2 and BMP15, through gene editing technology.	Highly simulates the genetic background of human POIs; enables research into the functions of specific genes.	Technically complex and costly; gene editing may produce off-target effects.	(150, 151)
Immune induction method	Transparent zone antigen immunology, thymectomy	Inducing autoimmune ovarian inflammation by immunizing with ZP protein or removing the thymus leads to ovarian dysfunction.	Simulate the immune mechanism of POI; study the effects of immunotherapy.	High cost and high technical requirements; poor model stability and high animal mortality rate.	(152)
Radiation-induced method	X-ray or gamma ray irradiation	Radiation damage to ovarian tissue leads to ovarian dysfunction.	Easy to operate, it can quickly induce ovarian function decline; it can be used to study the effects of radiation on the ovaries.	Radiation may affect other organs; model stability is poor, and there are large individual differences.	(153)
Environmental toxin induction method	4-Vinylcyclohexene dicarboxylic acid diester (VCD) induced	VCD damages ovarian oocytes, leading to ovarian dysfunction.	The model reflects the actual impact of environmental toxins on the ovaries; toxin dosage and exposure time can be precisely controlled.	Different animals have different sensitivities to toxins; the mechanisms of action of environmental toxins are complex; animal testing is involved, which raises ethical concerns.	(154)
Dietary induction method	High-fat, high-sugar diet induces	A long-term high-fat, high-sugar diet produces excessive reactive oxygen species (ROS) and advanced glycation end products (AGEs), which in turn affect ovarian function.	Dietary interventions are easy to implement and relatively inexpensive; simulate the effects of metabolic abnormalities on ovarian function.	Molding takes a long time; diet-induced POI involves multiple metabolic and endocrine changes, and the mechanism is complex.	(155)
Stress induction method	Chronic stress and oxidative stress	Induce ovarian function decline through simulated environmental or psychological stress.	Simulates clinical characteristics; relatively simple to operate.	Molding time is relatively long; model stability is insufficient.	(156)
Surgical induction method	Oophorectomy	Simulate ovarian insufficiency by surgically removing part or all of the ovarian tissue.	The model is stable and reproducible; it can accurately control the degree of ovarian function decline.	The surgery is traumatic and requires a long recovery period; it is not possible to simulate the natural course of the disease.	(157)
Natural aging model	Elderly animal model	Use naturally aging animals to simulate POI caused by aging in humans.	It is possible to observe the dynamic process of ovarian function changes with age.	High time costs; significant individual differences.	(158)

Currently, the most commonly used method in laboratories is chemical drug induction, which directly damages ovarian tissue or interferes with follicle development through drugs. This method is simple to perform and has a short modeling cycle. Intraperitoneal injection of cyclophosphamide can induce apoptosis of ovarian granulosa cells in mice, leading to increased follicle atresia and hormonal imbalances (such as elevated FSH, decreased AMH, and decreased estrogen levels), which meets the clinical characteristics of POI (61). Another method involves using oral administration of Tripterygium glycosides to disrupt mitochondrial function in follicular granulosa cells, inducing an imbalance in the B-cell lymphoma-2 (Bcl-2) / Bcl-2-associated X protein (Bax) ratio and activating the apoptotic signaling pathway, which is suitable for studying oxidative stress and apoptotic mechanisms (62). Studies have shown that when the modeling dose of Tripterygium glycosides is 40 mg/kg, the number of growing follicles and corpus luteum in rats decreases, and E2 and P levels decrease, but there are no significant changes in FSH, LH, PRL, and T levels (63). When the molding agent exceeds 70 mg/kg, rats exhibit prolonged or absent estrous cycles, irregular changes, or even complete disruption. FSH and LH levels are significantly elevated, whereas E2 levels are significantly reduced, indicating that a stable POI model has been successfully established (64, 65).

In the selection of experimental animal models, differences in genetic background and physiological characteristics among different strains of animals may lead to significant heterogeneity in research results (66). Literature analysis indicates that the C57BL/6J mouse strain is the most commonly used mouse strain in current POI research, characterized by a well-defined genetic background and strong reproductive capacity, making it suitable for various induction methods and experimental procedures. In chemically induced POI models, C57BL/6J mice are widely used to evaluate the efficacy of mesenchymal stem cell-derived extracellular vesicles in improving ovarian function. Another commonly used animal model in POI research is the ICR mouse strain. This closed-breed strain, characterized by high genetic diversity, demonstrates unique value in studies of environmental adaptability and phenotypic heterogeneity. In rat model systems, Sprague Dawley and Wistar strains are highly favored due to their good tolerance to drugs and chemicals, and their physiological characteristics are particularly suitable for constructing different types of POI models, such as chemically induced and radiation-induced models.

Nevertheless, an important limitation of current animal studies is the discrepancy in acupoint selection compared with clinical practice. Whereas clinical trials most frequently use acupoints such as Shenshu (BL23) and Ciliao (BL31), animal experiments typically employ Guanyuan (CV4) and Sanyinjiao (SP6) (67, 68). This inconsistency reflects differences in anatomical feasibility between humans and small animals, but it raises concerns about the translational validity of preclinical findings. Results derived from animal models may therefore not fully capture the therapeutic effects observed in clinical electroacupuncture protocols. Recognizing this gap early in the discussion underscores the need for more standardized and clinically aligned acupoint selection in animal studies to enhance the translational value of preclinical data.

### 4.2 Mechanism of action research

EA is a combination of traditional acupuncture and modern electrical stimulation technology. It involves applying low-frequency pulsed currents to specific acupoints to regulate the flow of qi and blood and the functions of the internal organs. In recent years, EA therapy has shown great promise in the treatment of gynecological diseases. Research indicates that EA can have a positive impact on patients with POI through a variety of mechanisms, including improving ovarian function, regulating the hypothalamic–pituitary–ovarian (HPO) axis, influencing the neuroendocrine–immune network, regulating inflammatory responses, regulating ovarian blood flow, influencing mesenchymal stem cell function, and affecting the intestinal microenvironment.

#### 4.2.1 The effect of EA on ovarian function

The ovaries are the site where female reproductive cells (eggs) are produced. From the embryonic stage onward, the ovaries contain a large number of primordial follicles, which constitute the main component of the ovarian reserve. Primordial follicles consist of a primordial oocyte in the prophase of meiosis and a layer of flat granulosa cells, and they remain in a dormant state. Over time, some primordial follicles are activated and enter the growth phase, eventually developing into mature follicles and releasing eggs. The number of primordial follicles is fixed at birth, and their activation process leads to an irreversible decline in reproductive capacity (69). As Zhang et al. showed (70), EA stimulation of the Guanyuan (CV4) and Zusanli (ST36) acupoints in POI mice increased the number of primordial follicles after 7 days of intervention. Mating experiments showed that their fertility was significantly improved compared with the control group.

However, not all activated follicles develop to maturity. Most follicles stop growing and degenerate for various reasons, a process known as follicular atresia. Increased follicular atresia reduces the number of follicles available for development, thereby affecting ovarian reserve function. Wang Weiming et al. used EA to treat rats with POI models. After 4 weeks of treatment, they found that the number of closed follicles had significantly decreased, alleviating ovarian damage (16). Another experiment selected the Guanyuan (CV4), Sanyinjiao (SP6), and Guilai (ST29) acupoints for EA stimulation. The results showed that the volume of the ovaries increased, the number of atretic cells decreased, the structure of the oocytes became more distinct, the number of granulosa cells increased and became more orderly, and the number and volume of corpus luteum improved (71).

Around the oocyte, ovarian granulosa cells provide nutrients to the oocyte through intercellular connections while secreting FSH and E2, playing a key role in follicle growth and development. Studies have shown that POI patients have abnormally elevated levels of autophagy in ovarian granulosa cells, leading to dysfunction and accelerated follicle atresia, ultimately affecting ovarian reserve and fertility (72). EA improves ovarian function by regulating granulosa cell autophagy. Zhang Yi et al. found that EA at the Gongsun (SP4) acupoint reduces the number of autophagosomes and autophagolysosomes, upregulates p62 expression, downregulates Beclin-1 and LC3 protein, inhibits granulosa cell autophagy, and thereby improves ovarian reserve in POI (73). The therapeutic effects of EA at the Gongsun (SP4) acupoint provide a reference for acupoint selection in the clinical treatment of POI and lay the experimental foundation for the application of the theory of the Eight Extra Meridians. Additionally, the antiapoptotic factor Bcl-2 and the pro-apoptotic factor Bax are key molecules regulating cell apoptosis. The experiment demonstrated that electroacupuncture can upregulate Bcl-2 expression and downregulate Bax expression, thereby promoting granulosa cell proliferation and antiapoptotic capacity, thus protecting the ovaries (74).

In summary, EA can protect primordial follicles in the ovaries, reduce the number of atretic follicles, and regulate autophagy and apoptosis in granulosa cells, thereby improving ovarian reserve and function.

#### 4.2.2 The effect of EA on the neuroendocrine–immune network

The nervous system, endocrine system, and immune system are closely interrelated. They all contain neurotransmitters, endocrine hormones, and cytokines, among other components, and exhibit cross-reactivity with one another. The network they form is referred to as the neuroendocrine–immune network. Previous studies have shown that EA at the Lianquan (CV23) acupoint can activate the M1 (primary motor cortex)–PBN (parabrachial nucleus)–NTS (nucleus tractus solitarius) neural circuit, increase the excitability of neurons in the PBN and NTS, and significantly improve the swallowing function of PSD mice (75). The level changes β-endorphin (β-EP), as an important endogenous neurotransmitter, are closely related to the occurrence of POI (76). Research has found that EAstimulation of acupoints such as Guanyuan (CV4), Sanyinjiao (SP6), and Baihui (GV20) in POI model rats significantly increases β-EP levels in peripheral blood and the hypothalamus, thereby improving ovarian function (77). In addition, EA can also regulate neurotransmitter metabolism levels in the liver and kidneys of POI mice. By regulating the levels of metabolites such as glutamate, taurine, glycine, and phosphatidylcholine, it restores the balance of neurotransmitter metabolism, thereby promoting the recovery of ovarian function (67). This discovery not only provides a new perspective on the pathogenesis of POI but also provides modern scientific evidence for the relationship between the causes of POI and liver and kidney function.

#### 4.2.3 The effect of EA on the HPO axis

The hypothalamus is the starting point of the HPO axis, regulating pituitary hormone secretion by secreting gonadotropin-releasing hormone (GnRH). Under the stimulation of GnRH, the pituitary gland secretes FSH and LH. FSH promotes follicle development and maturation, whereas LH promotes ovulation and corpus luteum formation, maintaining corpus luteum function. The ovaries, as the target organ of the HPO axis, are responsible for producing eggs and secreting sex hormones. Elevated levels of GnRH, FSH, and LH, along with decreased levels of E2 and AMH, are typical characteristics of HPO axis dysfunction (78). He Qi Da et al. performed electroacupuncture treatment on POI mice, and the results showed that serum FSH and LH levels were significantly reduced, whereas E2 and AMH levels were significantly increased, thereby regulating sex hormone levels (13). Another study found that EA stimulation of Baihui(GV20), Shenshu (BL23), and Sanyinjiao (SP6) can increase serum E2 levels in rats, decrease LH and GnRH levels, and restore the regulatory response between the ovaries and the pituitary gland (79). The above studies indicate that EA has a beneficial regulatory effect on the HPO axis by regulating hormone levels and protecting ovarian function.

#### 4.2.4 The effect of EA on inflammatory responses

Chronic inflammation of the ovaries is considered to be one of the important mechanisms underlying POI. Studies have shown that POI patients often have ovarian tissue infiltrated by large numbers of lymphocytes and macrophages, and elevated levels of proinflammatory factors such as tumor necrosis factor-α (TNF-α) and interferon-γ (IFN-γ) in the serum, whereas levels of anti-inflammatory factors such as interleukin-4 (IL-4) are significantly decreased (80). TNF-α plays a key role in regulating the proliferation and apoptosis of ovarian granulosa cells. Its overexpression can induce granulosa cell apoptosis, accelerate follicular atresia, and thereby impair ovarian function. Animal experiments have shown that EA intervention significantly downregulates TNF-a protein expression in mouse ovaries, reduces granulosa cell apoptosis, and promotes the recovery of ovarian function (71). In addition, EA combined with moxibustion can inhibit the expression of the pro-inflammatory factor IFN-g, while significantly upregulating IL-4 levels (81). IL-4 can antagonize the inflammatory effects of IFN-γ, induce Th0 cells to differentiate into Th2 cells, regulate the Th1/Th2 immune balance, improve the local inflammatory microenvironment, and thereby delay ovarian function decline. These research findings suggest that EA, by regulating the expression of proinflammatory/anti-inflammatory factors and intervening in immune homeostasis, has positive significance in inhibiting inflammation associated with POI.

#### 4.2.5 The effect of EA on ovarian blood flow

Vaginal ultrasound examination showed that POI patients had lower ovarian vascularization index, blood flow index, systolic peak flow velocity, and end-diastolic flow velocity than normal values, whereas pulsatility index and resistance index were significantly elevated. As ovarian function deteriorates, blood supply to the ovaries also gradually decreases (82). Research has confirmed that EA on acupoints such as Baihui (GV20) can effectively reduce the pulsation index of ovarian artery blood flow and improve ovarian blood perfusion (83). Animal experiments further revealed that the regulation of ovarian blood flow by EA is frequency- and current intensity-dependent: low frequency (2 Hz) combined with moderate intensity (3 mA, 6 mA) increases OBF, whereas high frequency (80 Hz) and high intensity (6 mA) decrease ovarian blood flow (84).

#### 4.2.6 The effect of EA on MSCs

MSCs are widely present in tissues such as bone marrow, adipose tissue, umbilical cord, and placenta and possess multidirectional differentiation potential and powerful paracrine functions. The mechanisms of MSCs therapy for POI include homing, migration, and colonization in damaged ovaries, followed by the secretion of various growth factors, cytokines, and extracellular vesicles to exert immunomodulatory, pro-angiogenic, antiapoptotic, and antioxidant effects (85). The homing of MSCs primarily depends on the interaction between chemokine receptors on the cell surface (such as CXCR4) and chemokines in tissues (such as SDF-1), and downregulation of CXCR4 expression is an important cause of low MSCs homing efficiency (86). Studies have shown that EA can upregulate CXCR4 protein expression, significantly promote the migration of BMSCs to ovarian tissue, and enhance their antiapoptotic effects (87).

#### 4.2.7 The effect of EA on intestinal microorganisms

The human gut harbors a vast array of microbial communities, including probiotics, pathogens, and opportunistic pathogens, which are closely associated with human health. Existing research has shown that the gut microbiome can secrete β-glucuronidase, which influences estrogen levels by binding to estrogen receptors (88). Dysbiosis of the gut microbiota and reduced diversity can decrease β-glucuronidase activity and lead to reduced levels of active estrogens. Pathogenic bacteria in the gut and their metabolic products can reach the ovaries via the bloodstream and affect ovarian function (89). Recent studies have shown that the gut microbiota structure of POI patients undergoes significant changes: The abundance of Firmicutes, Brucella, and Faecalibacterium decreases, whereas the abundance of Bacteroidetes and Bacteroides increases (90). Experiments have confirmed that EA can regulate the composition and diversity of the intestinal microbiota, increase the proportion of beneficial bacteria, reduce the abundance of harmful bacteria, improve the intestinal microenvironment, and influence ovarian function through the gut-ovary axis, providing a new mechanism for EA treatment of POI (13).

#### 4.2.8 Integrated mechanistic hypothesis

Although the above subsections present the effects of EA on ovarian function, the neuroendocrine–immune network, the HPO axis, inflammatory responses, ovarian blood flow, MSCs activity, and the gut microbiota separately, these mechanisms do not operate in isolation. Increasing evidence suggests that EA may exert its therapeutic effects through an interconnected, multilevel regulatory cascade.

EA-induced modulation of the gut microbiota can restore microbial composition and diversity, thereby increasing the abundance of beneficial bacteria and reducing pathogenic taxa. This microbial rebalancing attenuates systemic low-grade inflammation by reducing endotoxin burden and modulating intestinal barrier function (91). In addition, the AMH level is closely related to granulosa cell apoptosis and ovarian inflammation (92). Reduced systemic inflammation in turn alters the inflammatory milieu of the ovary, downregulating pro-inflammatory cytokines and upregulating anti-inflammatory mediators, thereby improving granulosa cell survival and follicular health. Through this anti-inflammatory effect, EA indirectly reshapes the neuroendocrine–immune network, a system where cytokines, neurotransmitters, and hormones constantly interact. Lower systemic and ovarian inflammation allows for a more balanced cross talk between immune signals and neuroendocrine pathways, reflected in increased β-EP release and normalized neurotransmitter metabolism. In addition, a considerable number of researchers have found that acupuncture can regulate the activity of certain areas of the brain, downregulate the expression of estrogen receptors (ERs) in the hypothalamus, and regulate the function of GnRH neurons (93, 94). Consequently, the HPO axis regains regulatory balance: serum FSH and LH levels decrease, whereas E2 and AMH increase, leading to improved folliculogenesis and ovarian reserve. Meanwhile, EA also promotes ovarian blood flow and enhances mesenchymal stem cell homing, further supporting tissue repair, angiogenesis, and antiapoptotic processes within the ovary.

Taken together, these findings support a plausible integrative model in which EA initially modulates the gut microbiota and systemic inflammation, which subsequently influences the neuroendocrine–immune network, restores HPO axis function, and ultimately improves ovarian reserve and fertility. This interconnected framework emphasizes that the endocrine, immune, nervous, and microbial systems are deeply interwoven, and EA may act as a multitarget intervention capable of restoring their systemic balance.

### 4.3 Mechanism research

**Figure 4:**
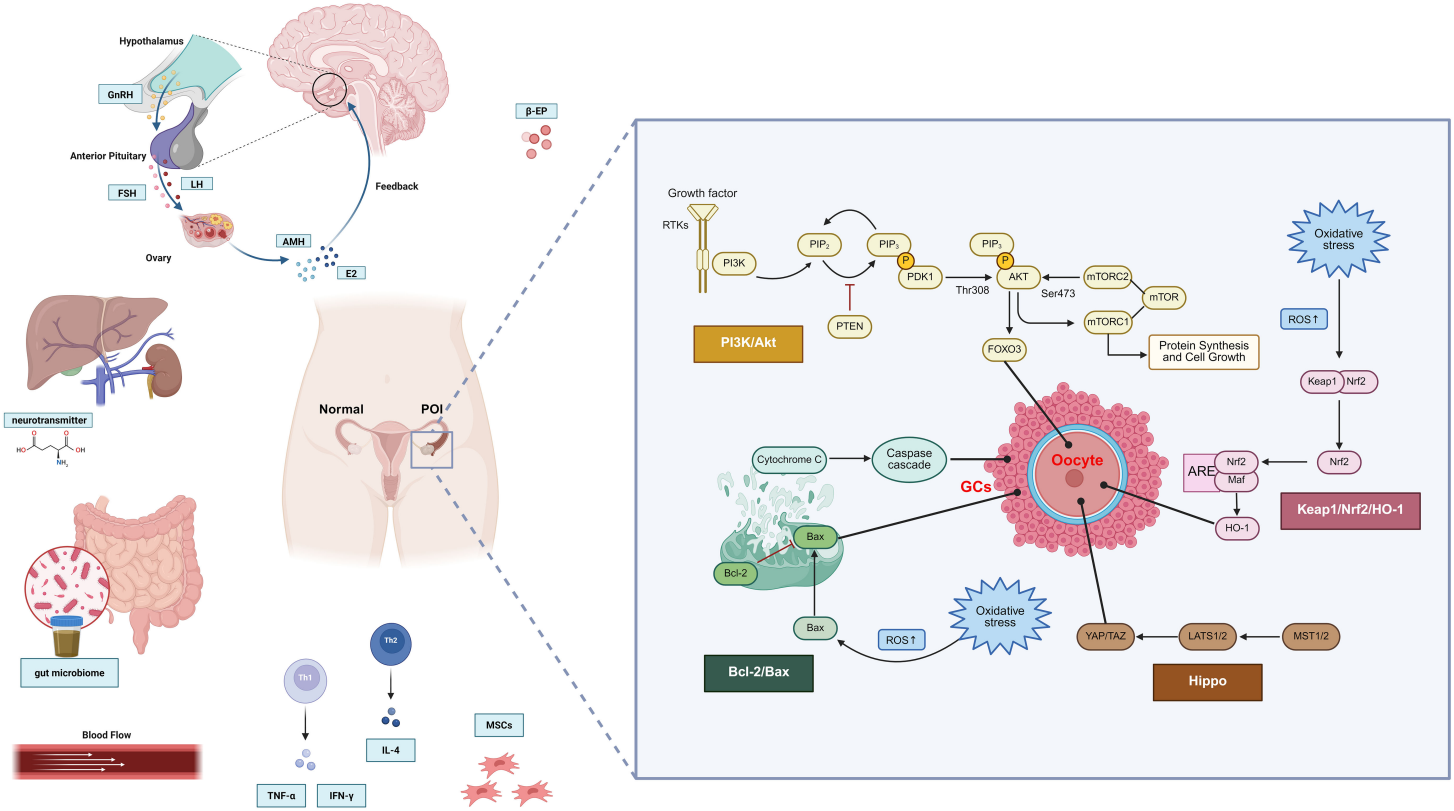
Signal pathways related to the occurrence and development of POI.

To better understand the pathophysiological process of POI, signaling pathways play a crucial role in the onset, progression, and treatment of POI. The loss of function of any molecule in these pathways may lead to a decline in ovarian function. In-depth research into these pathways can help elucidate the pathogenesis of POI and provide a theoretical basis for the development of new treatment methods (**Figure 4**).

#### 4.3.1 PI3K/Akt signaling pathway

Recent studies have shown that the phosphatidylinositol 3-kinase (PI3K)/protein kinase B (Akt) signaling pathway plays a central role in regulating follicle activation, survival, and ovarian aging. Before adulthood and puberty in females, most primordial follicles remain in a dormant state, and their dysfunction is one of the key molecular mechanisms underlying POI (95). This pathway maintains ovarian reserve by dynamically balancing the “resting-activated” state of follicles. Upon activation, receptor tyrosine kinases (RTKs) recruit intracellular PI3K to produce phospholipids. PI3K proteins belong to the lipid kinase family and can convert signals from various growth factors and cytokines into intracellular information, activating various downstream effectors (96). In the PI3K/AKT signaling pathway, PI3K phosphorylates phosphatidylinositol 2-phosphate (PIP2) to generate phosphatidylinositol 3-phosphate (PIP3), which accumulates on the cell membrane and initiates downstream signaling. 3-Phosphoinositide-dependent protein kinase-1 (PDK1) and Akt both contain PH domains, enabling them to specifically recognize and bind to PIP_3_. The accumulation of PIP_3_ promotes the translocation of these two proteins from the cytoplasm to the plasma membrane, bringing them into close spatial proximity and creating conditions for subsequent phosphorylation activation. The mammalian target of rapamycin (mTOR) is a downstream molecule of the PI3K/Akt pathway. mTORC1 and mTORC2 are two complexes of mTOR, which regulate cellular activities through different downstream targets. PDK1 and mTORC2 promote the phosphorylation of the threonine phosphorylation site Thr308 and the serine phosphorylation site Ser473, respectively, located in the regulatory domain of Akt. This phosphorylation is a necessary condition for the full activation of Akt (97). Activated AKT phosphorylates target proteins through multiple downstream pathways, thereby exerting an antiapoptotic effect.

Conflicting evidence has been reported regarding the effect of EA on this pathway. Zhang Han et al. demonstrated that EA significantly reduced the phosphorylation levels of PI3K, AKT, and mTOR in the ovaries of POI mice, suggesting that EA may prevent excessive primordial follicle activation and premature exhaustion of the ovarian reserve (70). This interpretation aligns with the hypothesis that hyperactivation of the PI3K/Akt/mTOR pathway accelerates follicular depletion, and its downregulation is protective. In contrast, other studies have reported the opposite. For instance, He QD et al. observed that EA increased the expression of PI3K, AKT, and mTOR in ovarian tissue, thereby promoting the proliferation of ovarian cells, ultimately improving ovarian function (13). Similarly, several acupuncture studies in other ovarian injury models have indicated pathway upregulation rather than inhibition. Taken together, these divergent findings suggest that the effects of EA on the PI3K/Akt/mTOR pathway may be context-dependent. Potential sources of discrepancy include the type of ovarian injury model (cyclophosphamide-induced *vs*. cisplatin-induced POI), the cell types examined (granulosa *vs*. oocytes *vs*. stromal cells), the stage of disease progression, and the specific acupoint selection and stimulation parameters. At present, the evidence is insufficient to draw a definitive conclusion, and further mechanistic studies are required to clarify whether EA primarily suppresses overactivation of dormant follicles or enhances survival pathways in granulosa cells, or whether both effects coexist depending on the physiological context.

#### 4.3.2 Hippo signaling pathway

The Hippo signaling pathway is a highly conserved pathway that was first thought to be a tumor-suppressor pathway, regulating tumor growth and organ size by inhibiting cell proliferation and promoting cell apoptosis (98). Current research indicates that the core components of the Hippo signaling pathway include mammalian Ste20-like protein kinase 1/2 (MST1/2), large tumor suppressor kinase 1/2 (LATS1/2), Yes-associated protein (YAP), and WW domain-containing transcription regulator 1 (WWTR1 or TAZ). These components are widely present in ovarian oocytes, granulosa cells, theca cells, and corpus luteum cells, and they influence various stages of follicle growth, playing a significant role in ovarian physiological functions. The activation of the Hippo pathway is regulated by multiple upstream signals. The upstream kinase MST1/2 activates LATS1/2, which further phosphorylates the transcription co-activator YAP/TAZ. Phosphorylated YAP/TAZ is retained in the cytoplasm and loses its transcriptional activity, whereas unphosphorylated YAP/TAZ enters the nucleus, activates the expression of downstream genes, and promotes cell proliferation (99). During the primordial follicle stage, the Hippo pathway maintains the resting state of the follicles by inhibiting the activity of YAP/TAZ, preventing their premature activation (100). Experimental data indicate that EA at the Guanyuan (CV4) acupoint can reduce granulosa cell apoptosis, enhance mitochondrial autophagy by inhibiting the Hippo-YAP/TAZ pathway, and thereby improve ovarian function (9). In addition, research by Philippe Godin et al. has confirmed that YAP1/TAZ also participates in the induction of multiple LH target genes (such as Areg, Pgr, and Ptgs2) in granulosa cells, thereby regulating the ovulation process (101).

#### 4.3.3 Cell apoptosis

Bcl-2/Bax proteins are key proteins in the regulation of apoptosis, primarily determining cell survival or death by regulating mitochondrial apoptosis. When cells are damaged, the pro-apoptotic protein Bax is activated, translocates to the outer mitochondrial membrane and forms pores, leading to the release of pro-apoptotic factors such as cytochrome C, activating the downstream caspase cascade reaction, and ultimately causing granular cell apoptosis (102). The antiapoptotic protein Bcl-2 inhibits apoptosis by binding to Bax, thereby suppressing its activity and preventing an increase in mitochondrial membrane permeability. In POI patients or animal models, Bax expression in ovarian tissue is typically significantly increased, whereas Bcl-2 expression is relatively reduced, leading to a decrease in the Bcl-2/Bax ratio. This change promotes apoptosis of granulosa cells and oocytes, accelerating follicle depletion. A study investigated the therapeutic effects of EA stimulation of the Zhongliao (BL33) and Tianshu (ST25) acupoints on POI mice. The results showed that after EA intervention, Bax expression in ovarian tissue was significantly reduced, whereas Bcl-2 expression was significantly increased. By regulating Bcl-2/Bax levels, apoptosis of granulosa cells was inhibited, follicle atresia was reduced, and normal follicle development was promoted (103). In addition, Bcl-2/Bax can also synergize with the Hippo pathway to jointly regulate cell survival and proliferation. Studies have shown that EA can enhance the antiapoptotic ability of granular cells by regulating the Hippo pathway and upregulating Bcl-2 and downregulating Bax expression, thereby reducing cell damage induced by oxidative stress and a high-fat, high-sugar diet (104).

#### 4.3.4 Oxidative stress

Oxidative stress is another key pathological mechanism underlying the onset and progression of POI. Its core manifestation is an imbalance between the production and clearance of ROS, leading to cytotoxic reactions such as lipid peroxidation, protein modification, and DNA damage. In POI patients, MDA levels in the ovaries are significantly elevated, whereas the activity of antioxidant enzymes (such as superoxide dismutase (SOD), glutathione peroxidase (GSHPx), and catalase (CAT)) and glutathione (GSH) is markedly reduced. This oxidative stress state can lead to mitochondrial DNA damage, disrupt the meiosis process of oocytes, and promote granulosa cell apoptosis, ultimately resulting in follicle atresia and reduced oocyte quality (105). It is worth noting that Fe²^+^ in the Fenton reaction can generate strong oxidative hydroxyl radicals (·OH), further exacerbating oxidative damage (106).

EA demonstrates significant advantages in regulating oxidative stress. Research has shown that EA intervention can effectively regulate the expression of Kelch-like ECH-associated protein 1 (Keap1)/nuclear factor erythroid 2–related factor 2 (Nrf2)/heme oxygenase-1 (HO-1) proteins, thereby enhancing the antioxidant capacity of ovarian tissue. Nrf2 is a key transcription factor in cellular responses to oxidative stress. Upon activation, it translocates to the cell nucleus, binds to small Maf proteins, and promotes downstream antioxidant response element (ARE)-mediated gene transcription, particularly the expression of heme oxygenase-1 (HO-1) (107). Keap1, as a negative regulator of Nrf2, undergoes conformational changes under ROS stimulation, releasing Nrf2 into the cell nucleus and thereby initiating the antioxidant stress mechanism. Animal experiments have shown that EA can upregulate the expression of Nrf2 and HO-1, reduce Keap1 levels, and enhance antioxidant stress capacity (108). In addition, EA can significantly increase GSH and SOD activity, reduce MDA and ·OH levels, and inhibit Fe2+ accumulation, thereby alleviating oxidative stress damage to the ovaries (68, 108).

More importantly, oxidative stress interacts with inflammatory responses. Excessive ROS can induce the release of inflammatory factors, exacerbating ovarian damage (109). Conversely, inflammatory signaling pathways (such as Wnt/β-Catenin) can also activate ROS production, triggering ovarian granulosa cell apoptosis and fibrosis (110). Therefore, EA plays an important role in delaying the progression of POI and protecting ovarian function by bidirectionally regulating oxidative stress and inflammatory responses.

#### 4.3.5 Other mechanism studies

Currently, although the number of studies on EA treatment for POI is limited, some research has already revealed its potential mechanisms of action and related signaling pathways. Studies have shown that following oxidative stress-induced damage, the expression of Sirtuin 1 (SIRT1) in ovarian cells is impaired, leading to the inhibition of its downstream factors, including peroxisome proliferator-activated receptor gamma coactivator 1-alpha (PGC-1a) and Nrf2, which in turn results in the accumulation of ROS and the induction of cell apoptosis. PGC-1a and Nrf2 deacetylation is inhibited, leading to the production of excessive ROS and subsequent induction of cell apoptosis (111). The “Zhibian To Shuidao” acupuncture technique can regulate the SIRT1/PGC-1α/Nrf2 pathway to inhibit oxidative stress reactions in POI rats, reduce follicle atresia, improve hormone levels, enhance ovarian reserve capacity, and thereby improve ovarian cell function (112). Additionally, Zhao Wei’s research team used moxibustion on the Guanyuan (CV4) and Sanyinjiao (SP6) acupoints to intervene in POI rats and found that moxibustion significantly increased cyclic adenosine monophosphate (cAMP)/protein kinase A (PKA)/cAMP response element-binding protein (CREB) pathway-related proteins, thereby activating steroid synthesis in granulosa cells and improving ovarian function in POI rats (113). During follicle development, the Notch pathway, the stem cell factor (Kit ligand, KL)-KIT proto-oncogene (KIT proto-oncogene, receptor tyrosine kinase, KIT) pathway, the transforming growth factor-beta (TGF-β)/Smad pathway, the Janus kinase (JAK)/signal transducer and activator of transcription (STAT) pathway, the mitogen-activated protein kinase (MAPK)/c-JUN/N-rosorbins (JUN/NRF) pathway, and others also play a role (114). Despite promising preclinical findings, current evidence on EA for POI remains preliminary. Most studies are limited to animal models with small sample sizes and heterogeneous protocols, whereas human RCTs are scarce. Moreover, mechanistic investigations often focus on isolated pathways rather than the broader multitarget regulatory network. Future studies should therefore emphasize standardized clinical trials and system-level approaches to better clarify both efficacy and underlying mechanisms.

## 5 Clinical research on EA treatment for POI

### 5.1 Comparison of different EA treatment protocols

Literature over the past 5 years (**Table 3**) indicates that Shenshu (BL23) and Ciliao (BL31) are among the most frequently selected acupoints in electroacupuncture clinical studies for POI. Other commonly used acupoints include Ganshu (BL18), Qihai (CV6), Guanyuan (CV4), Uterus (EX-CA1), Sanyinjiao (SP6), and Zusanli (ST36). These choices reflect the theoretical framework of traditional Chinese medicine, in which kidney deficiency and liver qi stagnation are considered central mechanisms in the pathogenesis of POI. Points such as Shenshu (BL23) and Guanyuan (CV4) are thought to tonify kidney qi, whereas Ganshu (BL18) is associated with regulating liver qi, and lower abdominal points such as Ciliao (BL32) and Qihai (CV6) are traditionally used to promote local qi and blood circulation in the reproductive system. Zusanli (ST36) and Sanyinjiao (SP6) are valued for their role in strengthening spleen and stomach function, thereby enhancing overall qi and blood production.

**Table 3:** Clinical studies on electroacupuncture treatment for POI.

Acupoint	EA parameters	Treatment frequency and duration	Combination therapy	Control group	Efficacy evaluation indicators	Sample size	References
Group A: acupuncture at Baihui (GV20), Benshen (BG13), etc.; group B: EA at Shenshu (BL23), Ciliao (BL32). The two groups of acupoints are used alternately.	Dense-sparse wave, leave the needles in for 20 minutes.	3 times a week for 6 months.	Medicined diet	HRT	FSH, AHM, E2, FSH/LH, AFC, uterine and ovarian volume, KI	60	(159)
EA at Ciliao (BL32).	Dense-sparse wave, leave the needles in for 30 minutes.	Once every other day, for 3 months.	HRT	HRT	Uterine receptivity (LIF, αVβ3, glycodelin), SDS, uterine CT imaging	58	(160)
EA at Baihui (GV20), Yintang (GV29), Guanyuan (CV4), Guilai (ST29), Sanyinjiao (SP6), etc.	Continuous wave, leave the needles in for 35 minutes.	Once every 3 days for 3 months.	HRT	HRT	FSH, AHM, E2, NK, CD4+T, CD8+T, CD4+T/CD8+T, KI, AEs	102	(120)
Multi-point combination therapy for different stages of the menstrual cycle.	Dense-sparse wave, frequency 2/15 Hz, intensity 1–5 mA, leave the needles in for 30 minutes.	3 times a week for 3 months.	/	HRT	FSH, LH, E_2_, AMH, AFC	150	(10)
EA at Shenshu (BL23), Ciliao (BL32), etc.	Frequency 2 Hz, intensity 2–3 mA, leave the needles in for 30 minutes.	28 sessions, 12 weeks in total.	/	HRT	FSH, AMH, E2, LH, FSH/LH, KI, menstrual condition, AEs	76	(121)
EA at Shenshu (BL23) and Baliao (BL31, BL32, BL33, BL34).	Dense-sparse wave, frequency 2/100 Hz, leave the needle in for 20–30 minutes.	3 times a week for 3months.	Traditional Chinese medicine	HRT	FSH, E_2_, AMH, AFC, RI, S/D, KI	60	(161)
Group A: Acupuncture at Baihui (GV20), Benshen (BG13), etc.; group B: EA at Shenshu (BL23), Ciliao (BL32). Use the two sets of acupoints alternately.	Dense-sparse wave, leave the needles in for 30 minutes.	Twice a week for 3months.	Traditional Chinese medicine	HRT	FSH, AHM, E2, LH, AFC, ovarian volume, TNF-α, IFN-γ, AEs	70	(162)
Group A: Acupuncture at Baihui (BG13), Zhongwan (CV12), etc.; group B: EA at Shenshu (BL23), Ciliao (BL32). The two groups of acupoints are used alternately.	Dense-sparse wave, frequency 2/100Hz, leave the needles in for 30 minutes.	3 times a week, for a total of 36 times.	/	acupuncture	FSH, AHM, E2, LH, FSH/LH, AFC, KI, SAS, brain MRI imaging	24	(163)
Group A: Acupuncture at Baihui (BG13), Shenting (BG24), etc.; group B: EA at Shenshu (BL23), Ciliao (BL32). The two groups of acupoints are used alternately.	Dense-sparse wave, alternating density for 1.5 s.	3 times a week for 6 months.	Traditional Chinese medicine	EA	FSH, E2, FSH/LH, pregnancy status	60	(164)
EA at Uterus (EX-CA1), Shenshu (BL23), and Ciliao (BL32); acupuncture at Benshen (BG13) and Shenmen (HT7).	Continuous wave, frequency 1 Hz, leave the needles in for 20 min.	12 times a month for 3 months.	Ovulation induction protocol	Acupuncture	bFSH, AHM, AFC, high-quality oocyte rate, high-quality embryo rate	70	(165)

From a modern biomedical perspective, preliminary findings suggest that EA at points such as Zusanli (ST36) and Sanyinjiao (SP6) may influence oxidative stress and inflammatory responses in the ovaries, potentially by regulating antioxidant systems such as GSH (115). Furthermore, clinical studies have applied a variety of EA stimulation parameters. One of the most commonly used waveforms is the density wave, which typically alternates between 1 and 100 Hz. Current intensity is generally adjusted to patient tolerance, and needles are usually retained for 20–30 min per session. The waveform, frequency, intensity, and duration of stimulation are key factors influencing treatment efficacy (116). Despite these general patterns, significant variability exists across studies, and very few trials have systematically compared different frequencies, intensities, or waveforms. Consequently, the question of optimal EA parameters remains unresolved.

Most studies employ a control group, usually HRT, given that HRT is the current standard of care for POI. This design facilitates comparisons but also limits the scope of evidence, since HRT has well-documented adverse effects, including potential risks for breast cancer, endometrial hyperplasia, and venous thromboembolism. We need to design more clinical trial groups, such as comparing EA alone with HRT, rather than comparing them in combination with other methods. This will more clearly demonstrate the therapeutic effect of EA and have greater clinical application value and persuasiveness. In addition, EA requires frequent in-person visits over several weeks or months, which may reduce compliance compared with oral medications. This compliance issue introduces another variable into trial design and complicates the interpretation of treatment outcomes.

Preliminary findings from small-scale studies suggest that EA may be associated with improvements in serum sex hormone levels, such as reducing FSH, LH, and the FSH/LH ratio, while increasing E2 and AMH, which is consistent with the conclusions of relevant systematic reviews and meta-analyses (117, 118). Some studies have also noted improvements in psychological well-being and quality of life. Exploratory mechanistic studies have begun to examine functional brain changes through resting-state fMRI, and immunological effects such as modulation of T lymphocytes, NK cells, and inflammatory factors.

However, a critical appraisal of the current evidence is warranted. Most published trials are single-center, involve small sample sizes, and are at high risk of bias due to inadequate blinding and unclear allocation concealment. The reliance on surrogate endpoints such as hormone levels and KI scores—particularly the KI, which is now considered outdated and poorly validated—limits the interpretability of outcomes. Importantly, few studies have assessed clinically meaningful endpoints such as live birth rate, ovarian reserve recovery, or long-term quality of life. The very high “effective rates” reported in some single-center studies (e.g., >90%) are difficult to interpret, as definitions of “effectiveness” are inconsistent and often based on composite or subjective criteria.

Taken together, the current clinical literature should be interpreted cautiously. While early findings suggest that EA may offer symptomatic and biochemical improvements in POI, robust conclusions regarding efficacy cannot yet be drawn. There is an urgent need for well-designed, multicenter randomized controlled trials with larger sample sizes, standardized EA protocols, validated outcome measures, and long-term follow-up.

### 5.2 Safety assessment of EA treatment for POI

EA integrates traditional acupuncture with modern electrical stimulation, aiming to enhance the therapeutic effect by applying adjustable frequencies and intensities. Theoretically, this combination allows for more reproducible and sustained stimulation compared with manual acupuncture. Importantly, existing clinical observations suggest that the addition of electrical stimulation does not markedly increase the risk of adverse events (119).

In comparative trials, EA groups have reported fewer side effects than HRT groups. For instance, Wu Jia observed that no major adverse events such as fainting, retained needles,broken needles, or significant hematomas occurred in the EA group, whereas participants in the HRT group reported mild headaches and breast tenderness (120). Similarly, Xiang Yue et al. found that minor gastrointestinal discomfort, rash, and nausea occurred more frequently in patients receiving HRT compared with those undergoing EA (121). Based on these findings, EA may represent a treatment with a relatively favorable safety profile.

One proposed advantage is that EA appears to promote estrogen synthesis by stimulating endogenous regulatory pathways, rather than supplying exogenous hormones. This mechanism could theoretically reduce the risk of overstimulation of estrogen-sensitive tissues such as the breast and endometrium, which is a concern with long-term HRT. Additionally, because acupuncture is non-pharmacological, it may avoid drug-related interactions and complications, offering an alternative for patients with contraindications to HRT.

Nevertheless, it is important to emphasize the limitations of current safety data. The majority of studies are based on relatively small sample sizes and short follow-up durations, which may not be sufficient to detect rare or long-term adverse events. Reporting quality is also inconsistent, with many studies lacking standardized definitions or systematic monitoring of side effects. Moreover, since many trials use HRT as the sole comparator, the relative safety of EA compared with other non-hormonal interventions remains unclear.

In summary, available evidence suggests that EA may be a safe and acceptable therapeutic option for patients with POI, especially those unable or unwilling to undergo HRT. However, the current body of literature is insufficient to draw definitive conclusions. Large-scale, rigorously designed clinical trials with standardized adverse event reporting and longer follow-up are necessary to confirm whether the safety advantages suggested in preliminary studies hold true in broader clinical practice.

## 6 Limitations and challenges

EA has emerged as a potential therapeutic approach for treating POI in recent years, demonstrating unique therapeutic value in both clinical practice and basic research. However, its widespread application still faces numerous critical challenges. In terms of mechanism of action, the effects of EA in treating POI exhibit significant multisystem synergistic characteristics, potentially exerting its effects through multiple pathways such as the vagus nerve–ovarian axis, HPO axis, and the emerging gut microbiota–ovarian axis. However, current basic research primarily focuses on isolated analyses of individual signaling pathways or local mechanisms, failing to systematically elucidate the spatiotemporal dynamic changes of different regulatory nodes (such as PTEN and FoxO3a). Additionally, there is a lack of research on cross-organ, multidimensional studies involving central nervous system components (such as arcuate nucleus kisspeptin neurons), peripheral immune cells (such as regulatory T cells, natural killer cells), and metabolic organs (such as adipose tissue). Notably, the onset and progression of POI are closely associated with epigenetic alterations such as DNA methylation, histone modifications, and abnormal non-coding RNA (122). Although EA has been proven to regulate Parkinson’s disease (123) and chronic pain (124), epigenetic markers in diseases such as these have been identified, but research into their epigenetic regulatory effects on genes related to ovarian function (such as FOXL2 and BMP15) remains largely unexplored. This knowledge gap significantly hinders our comprehensive understanding of the mechanisms underlying the effects of EA.

The issue of species differences in animal experiments should not be overlooked. Currently, most studies directly apply human acupoint localization methods to animal models, but there are significant differences between humans and experimental animals in terms of anatomical structure, surface projection area, and neural innervation. For example, the sympathetic innervation of rat ovaries primarily originates from the T10-L2 spinal ganglia (125). Humans, on the other hand, involve the T10-T11 segment (126). These differences in neural innervation may lead to shifts in stimulation targets when directly applying human acupoint localization methods. Clinically, the most commonly used acupoints for treating POI are Shenshu (BL23) and Ciliao (BL32), but animal experiments often select Guanyuan (CV4) and Sanyinjiao (SP6). This discrepancy is partly due to practical difficulties in technical manipulation—locating the Ciliao (BL32) acupoint in rats requires reference to the projection of the sacral foramen, which presents issues such as poor operational stability and low reproducibility in experiments, whereas Guanyuan (CV4) and Sanyinjiao (SP6) are relatively easier to precisely locate in rat models. Additionally, the ovarian physiological characteristics of different animal models differ significantly from those of humans. For example, the estrous cycle of mice is only 4–5 days (127), much shorter than the 28-day menstrual cycle in humans, but existing studies have rarely systematically compared the differences in the effects of EA across different species. This oversight may lead to serious misjudgments when directly extrapolating animal experiment results to clinical practice.

The limitations of animal models are also a key factor constraining the depth of mechanism research. Currently, most EA studies employ a single modeling method. For example, the cyclophosphamide-induced chemotherapy damage model primarily simulates iatrogenic ovarian dysfunction, the 4-vinylcyclohexene dioxide (VCD) model mimics age-related ovarian dysfunction by destroying primordial follicles, and gene-edited models (such as FoxO3a knockout mice) can spontaneously exhibit POI phenotypes. The mechanisms of ovarian damage vary significantly across these different models. However, most existing EA studies only compare the “EA group” with the “model control group,” lacking cross-model, multifactor experimental designs. This makes it difficult to determine whether EA has differentiated therapeutic effects on POI caused by different etiologies or whether it acts through certain common pathways. This limitation in research design directly impacts the clinical guidance value of experimental conclusions.

The most prominent issue in current clinical research is the insufficient sample size, with most trials enrolling fewer than 100 cases, and some studies even fewer than 30 cases. Such small-sample studies are not only susceptible to random factors, leading to reduced reliability of results, but also make it difficult to accurately assess the true efficacy and safety of EA. The POI patient population itself exhibits high heterogeneity, including diverse etiologies (such as genetic, immune-related, or iatrogenic causes) and significant variations in baseline hormone levels. With insufficient sample sizes, researchers often cannot conduct detailed subgroup analyses, making it difficult to precisely assess the therapeutic effects of EA on specific populations. This limitation directly impacts the clinical guidance value of research conclusions, making it challenging to provide robust evidence-based support for clinical practice.

In terms of the design and implementation of treatment protocols, existing research exhibits significant deficiencies in standardization. Key parameters of EA intervention, including waveform selection, frequency settings, stimulation intensity, and treatment schedules, have not yet been standardized based on evidence-based medicine. Low-frequency (2–10 Hz) and high-frequency (50–100 Hz) EA, commonly used in clinical practice, may exert their effects through different neurophysiological mechanisms. However, existing studies have failed to clearly distinguish their respective indications and lack systematic evaluations of the effects of different parameter combinations. More concerning is that most studies adopt fixed treatment protocols without dynamically adjusting or optimizing them based on patients’ real-time responses (such as the sensation of needle insertion or changes in hormone levels during treatment). This “one-size-fits-all” approach may significantly impair the stability and reproducibility of treatment efficacy. In contrast, clinical studies on traditional Chinese medicine treatment for POI typically select patients based on traditional Chinese medicine syndrome differentiation, whereas EA studies mostly rely on Western medical diagnostic criteria, failing to fully leverage the distinctive advantages of traditional Chinese medicine syndrome differentiation and treatment. This results in acupoint selection being largely limited to conventional points such as Shenshu and Ciliao, with a lack of personalized treatment protocols tailored to different syndromes.

The lack of diversity in the efficacy evaluation system is another issue that needs to be addressed urgently. Currently, the efficacy of EA treatment for POI is mainly evaluated based on serum FSH, E2, AMH, and other hormone indicators (128). This single biological marker evaluation method has obvious limitations. POI, as a complex reproductive endocrine disorder, involves dysfunction across multiple systems and organs in its pathophysiological process. Focusing solely on hormonal level changes fails to comprehensively reflect treatment efficacy. In fact, POI patients often exhibit systemic changes such as metabolic abnormalities (e.g., increased waist circumference, alterations in lipid and glucose levels) and dysregulation of signaling pathways (e.g., changes in the phosphorylation levels of key molecules such as PI3K, AKT, and mTOR), yet these critical indicators are frequently overlooked in current research. More importantly, POI is fundamentally a chronic disease, and treatment goals should not be limited to short-term improvements in hormone levels but should also focus on the long-term stability of ovarian function and the restoration of reproductive potential. Unfortunately, most existing EA studies have follow-up periods of only 3–6 months, with long-term tracking data exceeding 1 year being extremely rare. Assessments of hard endpoints such as pregnancy rates and live birth rates are severely lacking, significantly limiting the application and promotional value of EA in the field of assisted reproduction.

## 7 Future directions

Looking ahead, research on EA treatment for POI requires breakthrough progress in several key areas. At the mechanistic level, the integrated application of multi-omics technologies will yield significant breakthroughs. Single-cell sequencing technology can reveal the specific regulatory effects of EA on different cell populations within the ovaries (such as granulosa cells, oocytes, and stromal cells); spatial transcriptomics can elucidate the cellular interaction networks within the follicular microenvironment; and metabolomics can help clarify the pathways through which EA regulates systemic energy metabolism. By combining cutting-edge technologies such as tissue clearing and high-resolution *in vivo* imaging, researchers can even map the spatiotemporal dynamics of ovarian function reconstruction under acupuncture intervention, providing more precise regulatory targets for clinical treatment. In-depth epigenetics research is also crucial, particularly in exploring the regulatory effects of acupuncture on epigenetic features such as methylation patterns and chromatin accessibility of genes related to ovarian function.

Optimization and innovation in animal research are equally indispensable. Advanced technologies such as micro-CT and 3D ultrasound imaging should be utilized to establish more precise standards for locating acupoints in animals. Additionally, disease models that more closely mimic clinical conditions should be developed for different etiologies, such as chemotherapy-induced damage and autoimmune attacks. Additionally, we should actively explore combined application schemes of EA with other emerging therapies (such as mitochondrial transplantation and platelet-rich plasma therapy), which may offer new treatment options for patients with refractory POI. It is worth noting that whereas research using non-human primate animal models is costly, it plays a significant role in enhancing the clinical translational value of research outcomes.

Promoting high-quality multicenter clinical research, through collaboration between reproductive medicine centers and traditional Chinese medicine institutions, and conducting rigorously designed randomized controlled trials is an urgent priority for clinical research. Such studies should have an adequate sample size (no fewer than 200 cases) and be scientifically stratified based on different etiologies (such as genetic, immune-related, and iatrogenic) and traditional Chinese medicine syndromes (such as kidney qi deficiency, liver qi stagnation, and blood stasis) to obtain more targeted evidence of efficacy. Treatment plan design should adhere to the Standards for Reporting Interventions in Acupuncture Clinical Trials (STRICTA) guidelines. Acupuncture protocols should be reported in detail, including acupoint selection, needle insertion depth, EA parameters (such as frequency, intensity, and retention time), and treatment frequency. Adoption of STRICTA will enhance methodological rigor, improve reproducibility, and facilitate comparisons across studies. Additionally, establishing reasonable controls (such as sham acupuncture, herbal medicine controls, or thread implantation therapy) is crucial, as this facilitates objective assessment of the unique therapeutic value of electroacupuncture.

Establishing a long-term follow-up mechanism and a comprehensive multidimensional efficacy evaluation system is another research direction that urgently needs to be strengthened. Existing studies have overly focused on short-term changes in hormone levels, while paying insufficient attention to long-term outcome indicators such as reproductive outcomes (e.g., ovulation rate, and live birth rate), metabolic markers (e.g., insulin resistance and bone density), psychological status, and quality of life. Future studies should extend the follow-up time to more than 1 year. While observing changes in serum hormone levels, new biomarkers such as reproductive outcome indicators (such as ovulation rate and pregnancy rate), ovarian-specific miRNA, and intestinal flora characteristics should be introduced to construct a dynamic and comprehensive efficacy evaluation system to truly reflect the long-term benefits of electroacupuncture treatment.

Promoting data sharing and interdisciplinary collaboration is a key strategy for accelerating progress in this field. Building an open POI database for EA treatment, integrating clinical efficacy data, basic mechanism research results, and real-world treatment experience, and using artificial intelligence technology to identify potential efficacy predictors and treatment response markers will not only accelerate scientific discovery but also promote the establishment of international treatment standards. A global scientific research collaboration network will help gather treatment experience from different regions and evaluate the efficacy differences of electroacupuncture in different populations.

## 8 Conclusion

EA represents a promising adjunctive approach for POI management, with preliminary evidence supporting its effects on ovarian function, hormonal regulation, and symptom relief. Its multitarget actions and integration within the framework of traditional and modern medicine make it a potential contributor to personalized treatment strategies.

However, the current body of evidence is insufficient to establish EA as a definitive therapy. Clinical trials are generally small, are heterogeneous in design, and often lack robust endpoints such as fertility outcomes or long-term follow-up. Mechanistic insights, though valuable, remain largely preclinical and face challenges in translation to human physiology. Additionally, discrepancies between animal experimental protocols and clinical practice highlight the need for standardization.

Future research should prioritize multicenter, large-scale randomized controlled trials with standardized EA protocols, rigorous blinding, and comprehensive outcome assessment—including fertility, quality of life, and psychological well-being. Mechanistic studies integrating molecular biology, systems medicine, and advanced imaging techniques may help unravel the pathways through which EA influences ovarian function.

In summary, EA for POI should currently be viewed as a potential adjunctive therapy supported by preliminary but inconclusive evidence. With continued high-quality research, it may evolve into an evidence-based option within integrative reproductive medicine.
